# Intercropping Induces Changes in Specific Secondary Metabolite Concentration in Ethiopian Kale (*Brassica carinata*) and African Nightshade (*Solanum scabrum*) under Controlled Conditions

**DOI:** 10.3389/fpls.2017.01700

**Published:** 2017-09-29

**Authors:** Benard Ngwene, Susanne Neugart, Susanne Baldermann, Beena Ravi, Monika Schreiner

**Affiliations:** ^1^Leibniz Institute of Vegetable and Ornamental Crops, Großbeeren, Germany; ^2^Institute of Nutritional Science, University of Potsdam, Potsdam, Germany; ^3^Department of Crop and Animal Sciences, Humboldt University of Berlin, Berlin, Germany

**Keywords:** intercropping, indigenous leafy vegetables, nutrition security, secondary plant metabolites, *Brassica carinata*, *Solanum scabrum*

## Abstract

Intercropping is widespread in small-holder farming systems in tropical regions and is also practiced in the cultivation of indigenous vegetables, to alleviate the multiple burdens of malnutrition. Due to interspecific competition and/or complementation between intercrops, intercropping may lead to changes in plants accumulation of minerals and secondary metabolites and hence, alter nutritional quality for consumers. Intercropping aims to intensify land productivity, while ensuring that nutritional quality is not compromised. This study aimed to investigate changes in minerals and secondary plant metabolites in intercropped *Brassica carinata* and *Solanum scabrum*, two important African indigenous vegetables, and evaluated the suitability of this combination for dryer areas. *B. carinata* and *S. scabrum* were grown for 6 weeks under controlled conditions in a greenhouse trial. Large rootboxes (8000 cm^3^ volume) were specifically designed for this experiment. Each rootbox was planted with two plants, either of the same plant species (mono) or one of each plant species (mixed). A quartz sand/soil substrate was used and fertilized adequately for optimal plant growth. During the last 4 weeks of the experiment, the plants were either supplied with optimal (65% WHC) or low (30% WHC) irrigation, to test the effect of a late-season drought. Intercropping increased total glucosinolate content in *B. carinata*, while maintaining biomass production and the contents of other health related minerals in both *B. carinata* and *S. scabrum*. Moreover, low irrigation led to an increase in carotene accumulation in both mono and intercropped *S. scabrum*, but not in *B. carinata*, while the majority of kaempferol glycosides and hydroxycinnamic acid derivatives of both species were decreased by intercropping and drought treatment. This study indicates that some health-related phytochemicals can be modified by intercropping or late-season drought, but field validation of these results is necessary before definite recommendation can be made to stakeholders.

## Introduction

Mixed cropping systems are practiced in many regions of the world in an attempt to intensify land productivity, and leverage the positive effects of biodiversity and better use of resources (AFSA, 2016). This practice is widespread in small holder farming systems in tropical regions (Lithourgidis et al., 2011) and also serve as a strategy to reduce agricultural risks through on-farm diversification (Olasantan, 2007; IFAD, 2011), due to lack of appropriate social security systems and other conventional risk mitigation possibilities. Intercropping also leads to improvement in soil fertility, conservation of soil and water, enhancement of microclimate conditions (Ehrmann and Ritz, 2014), and plant protection (Khan et al., 2010). Intercropping may also influence accumulation of minerals and secondary metabolites and hence, alters the nutritional quality mediated by interspecific competition and/or complementation (Inal et al., 2007; Li et al., 2014; Tong et al., 2015).

When practicing intercropping, farmers generally aim for yield improvement and/or stability (Hauggaard-Nielsen et al., 2008). However, quality attributes should not be compromised – especially in vulnerable regions like in Sub-Saharan Africa (SSA). Research on intercropping in SSA has generally focused on grain legume/cereal mix (Raseduzzaman, 2016) to improve soil fertility, as legumes are known to fix atmospheric nitrogen and replenish soils of this mostly rare commodity, which benefits the intercropped cereal (staple) crop (Shen et al., 2012). In many parts of SSA nowadays, however, there is growing interest and focus on highly nutritious foods such as indigenous leafy vegetables (ILV) because of their potential to promote nutrition and health of the consumers (Yang and Keding, 2009; Cernansky, 2015; Baldermann et al., 2016).

Sub-Saharan Africa is the region of the world with the highest prevalence of malnutrition, with over 200 million people affected representing more than 20% of the population (FAO, 2015). Consequently, the United Nation’s Sustainable Development Goals include as their second target the end of hunger and achieving food security and improved nutrition (Lartey, 2015). We are therefore faced with not only the challenge to produce enough food, but also to develop strategies to provide adequate nutrition to the population. This includes increased intake of minerals, vitamins, as well as health related secondary plant compounds.

Interestingly, nutritious ILVs are presently in vogue in many communities in SSA, and gaining popularity among consumers. In Kenya for example, ILVs are sold in large supermarkets in Nairobi, and seed companies are increasingly paying attention toward breeding traditional varieties (Cernansky, 2015). In these communities where foods of animal origin are not readily available, mainstreaming these nutritious ILVs into food systems to alleviate the multiple burdens of malnutrition has often been recommended (Abukutsa, 2010; Noorani et al., 2016). Such attempts should, however, exploit the multifunctionality of a mixed cropping systems (AFSA, 2016) advocated in such communities (Olasantan, 2007), and consider the nutritional changes that vegetables may undergo under such a system.

Ethiopian kale (*Brassica carinata*) is a leafy vegetable indigenous to East Africa, and gaining popularity in SSA. It is being advocated in urban and peri-urban horticulture in many parts of the African continent as a more nutritive alternative to *Brassica oleracea* (exotic counterpart) (Ambrose-Oji, 2009). It belongs to the order Brassicales which is characterized by a specific group of secondary plant metabolites – the glucosinolates (Verkerk et al., 2009). It has been shown that some break down products of glucosinolates – especially the isothiocyanates – possess anticancerogenic (Lippmann et al., 2014), anti-inflammatory (Herz et al., 2016) or antidiabetogenic (Guzmán-Pérez et al., 2016) properties. In addition to their richness in glucosinolates, *B. carinata* like other ILVs is rich in minerals and vitamins (Yang and Keding, 2009), and other secondary metabolites (flavonoids, carotenoids, and chlorophylls) (Neugart et al., 2017) possessing anti-oxidative properties and thus have the potential as natural sources for reducing cellular oxidative damage, and suppression of various cancers and cardiovascular diseases (Uusiku et al., 2010). Odongo et al. (2017) in a recent study highlighted the chemopreventive potential of *B. carinata*.

In one of the very few studies in SSA on intercropping Brassicals, the suitability of intercropping *B. carinata* with other ILVs (slender leaf (*Crotalaria* spp.), cowpea (*Vigna unguiculata*), African nightshade (*S. scabrum*), and spider plant (*Cleome gynandra*) was investigated (Oseko et al., 2006). They concluded that *V. unguiculata* and *S. scabrum* were suitable for intercropping with *B. carinata*. Their study was, however, focused on physiological investigations, and no consideration was put on nutritional and/or health promoting attributes that might be affected by intercropping. Both *V. unguiculata* and *S. scabrum* are considered quite nutritious but research-based evidence on the nutritional richness of *S. scabrum* is even more scarce even though it is one of the most popular ILV in some parts of SSA (Onyango and Imungi, 2007). While intercropping of *B. carinata* and *S. scabrum* may contribute to food and nutrition security in this region, it is important to understand nutritional changes that may occur under such systems.

In addition to changes induced by intercropping, plant biochemistry can also be affected by environmental factors like drought (Lisar et al., 2012), which is a very common phenomenon in SSA, and projected to increases with climate change alterations (Burke et al., 2009). Therefore large areas of production of these vegetables are presently, and will in future be affected by drought. Drought stress induces accumulation of reactive oxygen species (ROS) (Hasegawa et al., 2000). The accumulation of ROS may act as signals for inducing ROS scavengers and other protective mechanisms (Prasad et al., 1994). Drought induced oxidative damage in the plant tissue is controlled by a combined action of enzymatic and non-enzymatic antioxidant systems. Carotenes (e.g., β-carotene) form an important part of the plant antioxidant defense system. Apart from their role as accessory pigments, carotenes are effective antioxidant and contribute in protecting and sustaining photochemical processes (Havaux, 1998). Carotene accumulation in the plant is consequently influenced by plant water status. In the present study our objective was therefore to investigate changes in nutritional quality (minerals and secondary plant compounds focusing on glucosinolates, flavonoids, carotenoids, and chlorophylls) in leaves of these two important African ILVs (*B. carinata* and *S. scabrum*) as affected by intercropping. Additionally the suitability of the intercropping combination for dryer areas (drought treatment) was assessed, and the effect of a late-season drought on carotene accumulation was tested. Due to the difficulties in accurately managing water and fertilizer levels in the field, we decided for a greenhouse experiment to simulate these conditions.

## Materials and Methods

### Planting, Experimental Setup and Growth Conditions

**FIGURE 1:**
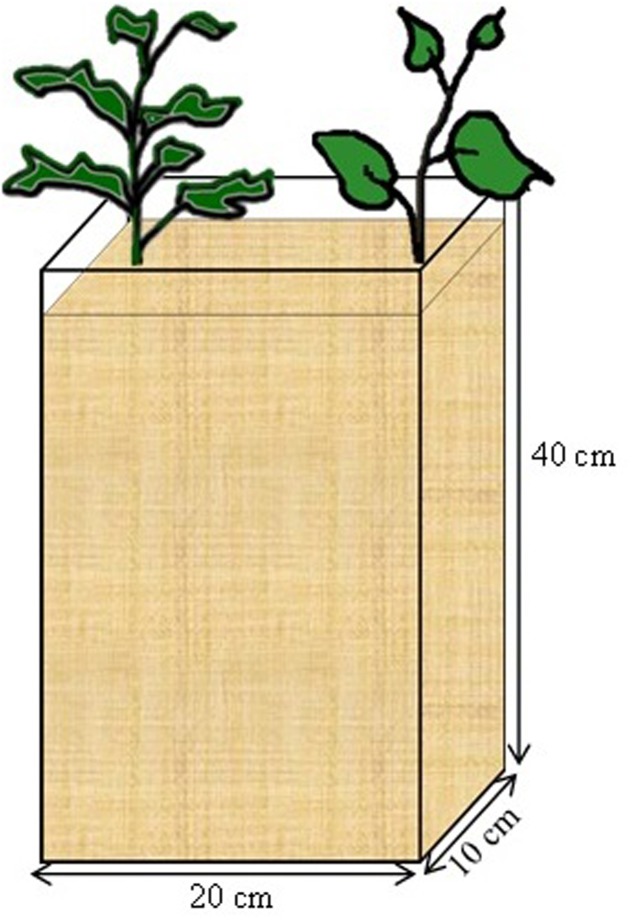
Rootbox constructed with 1 cm thick PVC plates (bottom and sides) and removable plexiglass plates (front and back). The internal dimension of the boxes was 20 cm (length), 10 cm (width), and 40 cm (depth) resulting in a total volume of 8000 cm^3^.

Seeds of *B. carinata* and *S. scabrum* obtained from the AVRDC Arusha, Tanzania were germinated in fine quartz sand for 1 week, when the primordial leaves were fully established. Two seedlings of the same crop (mono) or one of each crop (to simulate intercropping) were transplanted 15 cm apart in “rootboxes” (**Figure 1**). The rootboxes were constructed with 1 cm thick PVC plates (bottom and sides) and removable plexiglass plates (front and back). The internal dimension of the boxes was 20 cm (length), 10 cm (width), and 40 cm (depth) resulting in a total volume of 8000 cm^3^. Each rootbox was filled with a 50/50 v/v mixture of a soil substrate and coarse quartz sand. The soil substrate was a nutrient poor loamy sand, from the C-horizon of a luvisol, and had a pH of about 7.2. It was sieved through a 4 mm sieve, and fertilized with 200 mg N (NH_4_NO_3_), 100 mg P (KH_2_PO_4_), 400 mg K (K_2_SO_4_), 200 mg Mg (MgSO_4_), 20.8 mg Fe (Fe-EDTA), 20 mg Zn (ZnSO_4_), 20 mg Cu (CuSO_4_) kg^-1^ dry soil. This rate of fertilization was chosen based on previous experience with the soil substrate. The sides of the rootboxes were covered with an opaque plastic wrapping to prevent exposure to light. The rootboxes were arranged on a rack and inclined at 45°. The inclination was intended to encourage root growth toward and along the plexi-glass plates.

Water content in the mixed substrate was set at 65% water holding capacity (WHC) to ensure optimal water supply to the plants, and the plants were kept in a greenhouse. Water loss from the root-boxes was estimated gravimetrically and replaced regularly (once or twice daily). The experiment was set up in a completely randomized design and placed in a greenhouse in Grossbeeren, Germany (long. 13° 19′60′E; lat. 51° 22′0′N). The plants were grown under this irrigation regime for 2 weeks to well establish in the substrate before commencing of irrigation treatments. The irrigation treatments consisted of either maintaining optimal water supply (65% WHC) to half of the mono and mixed treatments, or simulating drought treatment (30% WHC) to the remaining half. The two factorial experiment therefore consisted of three cropping system treatments (mono *B. carinata*, mono *S. scabrum*, mixed *B. carinata*/ *S. scabrum*), and two irrigation treatments [65% (optimal supply) and 30% (drought simulation) WHC]. The plants were further cultivated for 4 weeks during which the plants were irrigated with an automatic irrigation system (GARDENA Urlaubsbewässerung-Set; GARDENA Manufacturing GmbH, Ulm, Germany), supplemented with gravimetric measurements. During cultivation, average day/night temperatures were 25°C/20°C, and relative humidity ranged from 52 to 83% with an average of 68%. The experiment was conducted between May and July, and all treatments had four replicates.

Within the first 2 weeks of cultivation, root growth was monitored after every 4 days by tracing roots growing along the plexi-glass plates with a permanent marker. A different color was used for each measurement. The length of roots growing along the plexi-glass plates at each measurement was latter estimated using a digital map reader (Wayfinder MR H; Huger Electronics GmbH, Germany).

### Harvest

Plants were harvested 6 weeks after transplanting. For each plant, all leaves were separated from the stem, the fresh weight (FW) was recorded, and frozen immediately. The frozen leaf material was later lyophilized at -30° for 1 week (CHRIST ALPHA; Martin Christ Gefriertrocknungsanlagen GmbH, Osterode am Harz, Germany), ground and stored for subsequent mineral and metabolite analyses. All other plant material were dried in an oven at 65°C for 3 days and the dry weight (DW) was noted.

### Nutrient Analysis

Sub samples of about 200 mg of the pulverized leaf material were digested with concentrated (65%) nitric acid and 30% hydrogen peroxide (5 and ml, respectively) for 15 min at 200°C in a microwave (MARSXpress 250/50; CEM Corporation, Matthews, NC, United States) and taken up to 25 ml with distilled water. After filtration (Filter circles MN 615, Macherey-Nagel Germany), P concentration in the filtrate was analyzed colorimetrically with a spectrophotometer (EPOS 5060, Eppendorf, Hamburg Germany) at 436 nm wavelength after staining with ammonium-molybdate vanadate solution (Gericke and Kurmies, 1952). Ca, Mg and Zn concentrations were measured by atomic absorption spectrometry (AAS; ATI Unicam 939/Solaar, Thermo Electron, United States).

### Secondary Plant Metabolite Analysis

#### Glucosinolate Analysis

Glucosinolates in lyophilized powdered leaf sample were assessed as desulfo-glucosinolates by using the method according to Wiesner et al. (2013) with slight modifications described in Omondi et al. (2017).

#### Flavonoid and Hydroxycinnamic Acid Analysis

To analyze the phenolic compounds, 20 mg lyophilized powdered leaf sample was extracted according to Neugart et al. (2015). For the quantitative analysis of flavonoid glycosides and hydroxycinnamic acid derivatives, an HPLC series 1100 (Agilent Technologies, Waldbronn, Germany) was used as described in Neugart et al. (2017). Standards (chlorogenic acid, quercetin 3-glucoside, kaempferol 3-glucoside and; isorhamnetin-3-glucoside Roth, Karlsruhe, Germany) were used for external calibration curves in a semi-quantitative approach. Results are presented in mg g^-1^ DW.

#### Carotenoid and Chlorophyll Pigments Analysis

Carotenoids and chlorophyll pigments were extracted from 5 mg of lyophilized ground leaves with methanol tetrahydrofuran (MeOH:THF) solution (1:1 v:v, 500 μL, 3 times) until colorless by shaking at 1000 rpm for 5 min and followed by centrifugation at 4500 rpm for 5 min. The combined supernatants were evaporated in a stream of nitrogen. The extracts were dissolved in 180 μL isopropyl alcohol and 20 μL dichloromethane. Prior to analysis the solutions were filtered through a 0.2 μM polytetrafluoroethylene (PTFE) membrane and kept at 4°C during the analysis process. An Agilent Technologies 1290 Infinity UHPLC equipped with a diode array detector and 6230 TOF LC/MS with an APCI ion source was used for the analysis. The gas temperature was set to 325°C at a flow rate of 8 L min^-1^. The nebulizer and the vaporizer pressure was set to 35 psi at a temperature of 350°C. The ionization voltage was set to +3500 V and a fragmentor voltage of 175 V was applied at a corona current of 6.5 μA. The separation was performed on a C30-stationary phase (YMC-Carotenoid, 100 mm × 2.1 mm, 3 μm YMC Co., Ltd., Japan) in gradient mode at a flow rate of 0.2 ml min^-1^. The mobile phases were composed of methanol, methyl-tert-butyl-ether and water in different volume ratios (solvent A: 81/15/4 and solvent B: 6/90/4). Ammonium acetate (20 mM) was added to the mobile phases to enhance the ionization. Identification was achieved based on retention times, absorption spectra and m/z of the pseudo molecular ions as compared with authentic reference substances. External standard calibration was achieved by dose–response curves.

### Statistical Analysis

All data were subjected to a two-way analysis of variance (ANOVA). The means were separated by a Tukey’s HSD test (*P* ≤ 0.05). Values obtained from the two plants in each monoculture were averaged for each replicate before statistical analysis was performed (*n* = 4). Statistics were performed using the Statistica software (Statistica 13; Dell Inc., United States)

## Results

### Plant Growth

Shoot DW of both *B. carinata* and *S. scabrum* were not significantly influenced by the cropping system, but by the irrigation treatment (**Table 1**). In the treatment supplied with low irrigation (W_low_), shoot DW was reduced significantly in both crops. There was no distinct effect of intercropping on the total yield in both optimal irrigated (W_opt_) and low irrigated treatments. There was also no difference in root length growing along the plexi-glass plates during the first 2 weeks of the experiment (data not shown).

**Table 1:** Shoot dry weight of *B. carinata* and *S. scabrum* at harvest (g/plant).

		Shoot dry weight (g)		Significant tests		
Crop	Irrigation	Mono	Mixed	*C*	*I*	*C* ×*I*
*B. carinata*	W_opt._	9.12 ± 0.48b	8.28 ± 1.03b	ns	^∗^	ns
	W_low_	6.31 ± 0.68a	5.87 ± 0.68a			
*S. scabrum*	W_opt._	8.16 ± 0.51a	9.22 ± 2.45a	ns	^∗^	ns
	W_low_	6.31 ± 0.64a	6.41 ± 1.24a			

*Two plants of the same crop (Mono) or one of each crop (Mixed) were grown in “rootboxes” and supplied with optimal (W_*opt.*_) or low (W_*Low*_) irrigation. Shown are the mean values (*n* = 4) obtained for each treatment ± standard deviation. Values in a column followed by the same letter are not significantly different (Tukey HSD test; *P* ≤ 0.05). The influence of the intercropped plant (*C*), irrigation level (*I*) and the interaction between both factors (*C* ×*I*) estimated by a two-way ANOVA are also presented. ns: not significant; ^∗^: significant at *P* ≤ 0.05*.

### Concentration of Mineral Nutrients

Similar to shoot growth, cropping system did not have a significant effect on mineral nutrient concentration in leaves except for Zn concentration in *B. carinata*. Irrigation, however, significantly influenced leaf concentration of Ca and P in *B. carinata*, and Mg, P and Zn in *S. scabrum* according to the two-way ANOVA (**Table 2**). The individual effects (direct comparison with their well-watered counterparts) where, however, not very strong (**Table 2**).

**Table 2:** Concentration of Ca, Mg, P and Zn in *B. carinata* and *S. scabrum* leaves at harvest.

		Ca (mg/g)		Mg (mg/g)		P (mg/g)		Zn (μg/g)	
Crop	Irrigation	Mono	Mixed	Mono	Mixed	Mono	Mixed	Mono	Mixed
*B. carinata*	W_opt._	31.97a ± 2.9	33.27a ± 3.6	6.24a ± 0.43	5.93a ± 0.51	2.85a ± 0.10	2.95a ± 0.19	137.2a ± 5.3	112.2a ± 15.2
	W_low_	30.42a ± 3.0	27.62a ± 2.9	6.14a ± 0.37	5.69a ± 0.13	2.55a ± 0.17	2.56a ± 0.34	136.7a ± 10.6	126.6a ± 17.4
*S. scabrum*	W_opt._	23.92a ± 7.0	21.06a ± 3.0	5.99ab ± 1.36	5.61a ± 0.85	2.83a ± 0.33	2.83a ± 0.12	158.4b ± 20.7	151.4b ± 7.4
	W_low_	22.76a ± 1.6	23.85a ± 2.8	6.76ab ± 0.47	7.32b ± 0.67	2.49a ± 0.14	2.66a ± 0.28	115.3a ± 10.8	135.6ab ± 12.9

**Significant tests for *B. carinata***									

Cropping (*C*)		ns		ns		ns		^∗^	
Irrigation (*I*)		^∗^		ns		^∗^		ns	
*C* ×*I*		ns		ns		ns		ns	

**Significant tests for *S. scabrum***									

Cropping (*C*)		ns		ns		ns		ns	
Irrigation (*I*)		ns		^∗^		^∗^		^∗^	
*C* ×*I*		ns		ns		ns		ns	

*For treatment description, see **Table 1**. Shown are the mean values (*n* = 4) obtained for each treatment ± standard deviation. Values in a column followed by the same letter are not significantly different (Tukey HSD test; *P* ≤ 0.05). The influence of the intercropped plant, irrigation level and the interaction between both factors estimated by a two-way ANOVA are also presented. ns: not significant; ^∗^: significant at *P* ≤ 0.05*.

### Concentration of Secondary Plant Metabolites

#### Glucosinolate

As glucosinolates are secondary plant metabolites which are limited to the order Brassicales, they were identified only in *B. carinata*.

**FIGURE 2:**
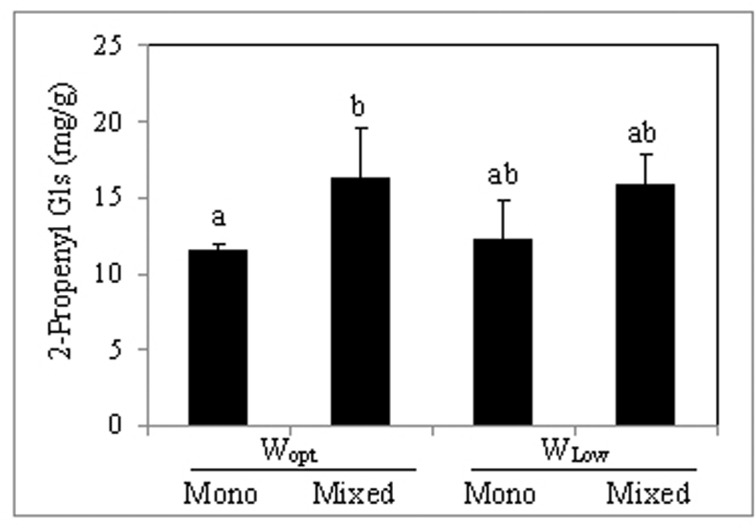
2-Propenyl glucosinolate concentration in *B. carinata* leaves (mg/g dry weight). For treatment description, see **Table 1**. Shown are the mean values (*n* = 4) obtained for each treatment + standard deviation. Bars followed by the same letter are not significantly different (Tukey HSD test; *P* ≤ 0.05).

The predominant glucosinolate in the leaves was 2-propenyl glucosinolate and it was the only detected aliphatic glucosinolate. Leaf concentration of 2-propenyl glucosinolate contributed to over 95% of total glucosinolate and was significantly increased by intercropping under optimal irrigation conditions (**Figure 2**) compared to sole cropping. Also the indole 3-indolylmethyl glucosinolate was quantitatively determined as well as its derivatives were assessed, but only in trace concentrations (**Table 3**). The concentration of 4-Hydroxy-3-indolylmethyl glucosinolate and 4-methoxy-3-indolylmethyl glucosinolate were also significantly increased by intercropping compared to sole cropping, while neither intercropping nor the variation in water supply conditions showed an effect on their precursor, 3-indolylmethyl glucosinolate, and the 1-methoxy-3-indolylmethyl glucosinolate (**Table 3**).

**Table 3:** Indole glucosinolate concentration in *B. carinata* leaves at harvest.

	I3M (mg/g)		4OHI3M (mg/g)		1MOI3M (mg/g)		4MOI3M (mg/g)	
Irrigation	Mono	Mixed	Mono	Mixed	Mono	Mixed	Mono	Mixed
W_opt._	0.15a ± 0.03	0.18a ± 0.03	0.09a ± 0.01	0.15ab ± 0.05	0.017a ± 0.001	0.024a ± 0.005	0.012b ± 0.004	0.007ab ± 0.005
W_low_	0.12a ± 0.02	0.16a ± 0.04	0.13ab ± 0.05	0.18b ± 0.03	0.021a ± 0.006	0.023a ± 0.006	0.008ab ± 0.005	0.003a ± 0.002

**Significant tests**								

Cropping (*C*)	ns		^∗^		ns		^∗^	
Irrigation (*I*)	ns		ns		ns		ns	
*C* ×*I*	ns		ns		ns		ns	

*For treatment description, see **Table 1**. Shown are the mean values (*n* = 4) obtained for each treatment ± standard deviation. Values in a column followed by the same letter are not significantly different (Tukey HSD test; *P* ≤ 0.05). The influence of the intercropped plant, irrigation level and the interaction between both factors estimated by a two-way ANOVA are also presented. ns: not significant; ^∗^: significant at *P* ≤ 0.05. I3M, Indolyl-3-methyl GLS; 4OHI3M, 4-hydroxy-3-indolylmethyl GLS; 1MOI3M, 1-methoxy-3-indolylmethyl GLS; 4MOI3M, 4-methoxy-3-indolylmethyl GLS*.

#### Carotenoids

**FIGURE 3:**
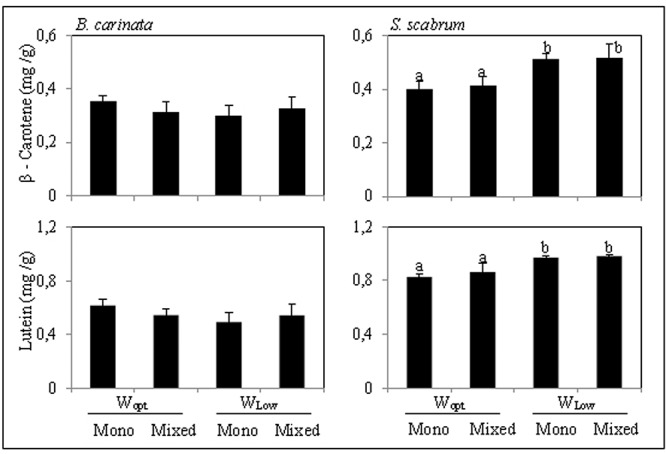
β-Carotene and lutein concentration in *B. carinata* and *S. scabrum* leaves (mg/g dry weight). For treatment description, see **Table 1**. Presented are the mean values (*n* = 4) obtained for each treatment + standard deviation. Bars followed by different letters are significantly different (Tukey HSD test; *P* ≤ 0.05).

In both crop species, β-carotene and lutein where the major carotenoids found. In *B. carinata* leaves, the concentration of both β-carotene and lutein were not influenced by either cropping system or irrigation treatment. In *S. scabrum* leaves, however, the concentration of both β-carotene and lutein were highly affected by the irrigation treatment (two-way ANOVA, *p* < 0.001 in both cases). There were consistently higher in the W_low_ plants compared to the corresponding W_opt_ plants (**Figure 3**).

#### Chlorophyll A and B

In *B. carinata* leaves, chlorophyll A concentration was not affected by cropping system or level of irrigation. However, the concentration of chlorophyll B was affected by the level of irrigation, and there was an interaction between cropping system and irrigation treatment. Low irrigation significantly increased chlorophyll B concentration as compared to optimal irrigation (**Table 4**). In *S. scabrum* leaves the concentration of both chlorophyll A and B was affected by level of irrigation, with higher values for the W_low_ treatment as compared to the W_opt_ treatment. Additionally, there was an interaction between the two factors on the concentration of chlorophyll B (**Table 4**).

**Table 4:** Chlorophyll A and B concentration in *B. carinata* and *S. scabrum* leaves at harvest.

		Chlorophyll A (mg/g)		Chlorophyll B (mg/g)	
Crop	Irrigation	Mono	Mixed	Mono	Mixed
*B. carinata*	W_opt._	1.33 ± 0.06a	1.11 ± 0.27a	0.64 ± 0.12b	0.54 ± 0.04ab
	W_low_	1.01 ± 0.30a	1.01 ± 0.24a	0.51 ± 0.07a	0.54 ± 0.08ab
*S. scabrum*	W_opt._	1.40 ± 0.12a	1.68 ± 0.17ab	0.62 ± 0.04a	0.67 ± 0.04a
	W_low_	1.89 ± 0.24b	1.78 ± 0.24ab	0.83 ± 0.01b	0.80 ± 0.03b

**Significant tests *B. carinata***					

Cropping (*C*)		ns		ns	
Irrigation (*I*)		ns		^∗^	
*C* ×*I*		ns		^∗^	

**Significant tests *S. scabrum***					

Cropping (*C*)		ns		ns	
Irrigation (*I*)		^∗^		^∗^	
*C* ×*I*		ns		^∗^	

*For treatment description, see **Table 1**. Shown are the mean values (*n* = 4) obtained for each treatment ± standard deviation. Values in a column followed by the same letter are not significantly different (Tukey HSD test; *P* ≤ 0.05). The influence of the intercropped plant, irrigation level and the interaction between both factors estimated by a two-way ANOVA are also presented. ns: not significant; ^∗^: significant at *P* ≤ 0.05*.

#### Flavonoids

The flavonoid glycosides and hydroxycinnamic acid derivative profiles of *B. carinata* and *S. scabrum* differ enormously (Neugart et al., 2017). *B. carinata* mainly has mono-alkylated kaempferol and isorhamnetin glycosides whereas *S. scabrum* contains mainly non-acylated quercetin glycosides. In *B. carinata* 13 out of 21 compounds mainly kaempferol glycosides were significantly reduced by intercropping. Additionally, 13 out of 21 compounds were also reduced by the lower irrigation level, and were mainly hydroxycinnamic acid derivatives and kaempferol glycosides. There was only one interaction between intercropping and irrigation level for 3-caffeoylquinic acid (Supplementary Tables S1A–C). In contrast, in *S. scabrum* only 3 out of 22 compounds were significantly reduced by intercropping whereas 14 compounds mainly hydroxycinnamic acid derivatives were reduced by W_low_ compared to W_opt_ treatment, but no interaction was observed (Supplementary Tables S1D–F).

## Discussion

### Plant Growth and Mineral Element Uptake

Intercropping had no effect on biomass production or mineral nutrient uptake and no clear belowground competition/complementation for mineral nutrient uptake was evident under our conditions. Plant growth (shoot biomass) was not affected by intercropping in both *B. carinata* and *S. scabrum*. While many studies have recorded yield improvement in mixed cropping systems (Shen et al., 2012; Wang et al., 2014; Raseduzzaman, 2016), related mostly to more efficient use of belowground resources, some studies have also reported no yield change (Oseko et al., 2006), or even yield reduction (Wang et al., 2007). These different outcomes are mostly due to the level of compatibility of the crops involved, and spatial and temporal availability of nutrients (Ehrmann and Ritz, 2014).

Most of the resources essential for plant growth are found belowground hence the main ecological processes defining the outcome of multiple cropping systems are the belowground competition for space and resources (Martin and Snaydon, 1982). Root distribution in the substrate (Zarea et al., 2009), and interactions therefore play a very important role in multiple cropping systems (Ehrmann and Ritz, 2014). In the present experiment, we did not observe any competition or complementarity between the *B. carinata* and the *S. scabrum* in terms of nutrient availability and use. This is indicated first in the results of root growth within the first part of the experiment, and second in the result of mineral nutrient uptake, where no difference was observed between sole- and inter-crops. The situation might be different in field grown crops where nutrient distribution and dynamics is not so homogeneous. Nevertheless, a similar result was obtained in the field by (Oseko et al., 2006) in intercropped *B. carinata* and *S. scabrum*.

While no competition for mineral nutrients was evident between the intercrops in this study, it is likely that there was adequate supply of nutrient in the substrate to support both plants. In conditions of nutrient limitation, mobilization processes are greater because of plant response and alteration of interspecific competition (Inal and Gunes, 2008). However, even when competition for nutrients is not evident in intercropping, there is nevertheless alteration in biological and chemical process in the rhizosphere (Inal et al., 2007) which can lead to changes in plant biochemistry.

In the absence of growth improvement though, the multifunctionality of a mixed cropping systems (AFSA, 2016) still prevails, as it ensures yield stability, diet diversify, and consequently improved nutrition (Frison, 2016; Uauy, 2016).

### Changes in Leaf Glucosinolates Concentration in *B. carinata*

The prevalence of the 2-propenyl glucosinolate in the glucosinolate profile of *B. carinata* was also demonstrated by other *B. carinata* accessions (Schreiner et al., 2009) suggesting the dominance of this aliphatic glucosinolate as specific characteristic of *B. carinata*. However, the *B. carinata* accession in the presented study (arumeru, Tanzania) was unaffected by reduced water supply, which is in contrast to other *B. carinata* accessions originating from Ethiopian (Holeta-1 and 37-A), and showing a distinct accumulation in 2-propenyl glucosinolate in the leaves under drought (Schreiner et al., 2009). Also in *B. juncea*, a *Brassica* species with 2-propenyl glucosinolate as major glucosinolate, topsoil drying caused a clear increase in the concentration of the predominant 2-propenyl glucosinolate in leaves and roots (Tong et al., 2014).

It could be assumed that there might be a *Brassica* species-specific as well as *B. carinata* accession-specific sensibility to the reduction of water supply suggesting a genotypic variation in the responsiveness to the drought-induced formation of abscisic acid as root-to-shoot signaling for increased aliphatic glucosinolate biosynthesis (Tong et al., 2014). This assumption is supported by Chen et al. (2012) who confirmed that the glucosinolate metabolism is linked to the plant’s hormone network including abscisic acid.

As seen in the aliphatic 2-propenyl glucosinolate, it seems that also all individual indole glucosinolates are not participating in plant responses to abiotic stress such as drought. They showed no response to the variation in the water supply conditions, which again is partly adversative to the drought reaction of the Ethiopian *B. carinata* accessions (Schreiner et al., 2009) and topsoil dried *B. juncea* (Tong et al., 2014). In these drought stress studies the 3-indolylmethyl glucosinolate in leaves increased at severely reduced soil water content in *B. carinata* accessions or the indole glucosinolates 3-indolylmethyl and 4-methoxy-3-indolylmethyl decreased under topsoil drying in *B. juncea*. However, all other indole glucosinolates were unaffected by the soil water conditions.

As found in our study in *B. carinata*, limited water supply did not affect indole glucosinolates in *B. rapa* ssp. *rapifera* (Zhang et al., 2008), whereas it increased indole glucosinolate concentration in leaves of *B. oleracea* (Radovich et al., 2005) and decreased it in *B. oleracea* var. *italica* (Robbins et al., 2005), suggesting genotypically different responses to drought also in respect to indole glucosinolates.

By intercropping an indigenous brassicaceous species (*B. carinata*) with a solanaceous species (*S. scabrum*), we specifically addressed the question of whether interactive effects of intercropping on the glucosinolate status in *B. carinata* occur under controlled condition. The aliphatic 2-propenyl glucosinolate distinctly increased under optimal water supply when intercropped with *S. scabrum*. Also the indole glucosinolate 4-hydroxy-3-indolylmethyl increased by intercropping under optimal water conditions, but in a less pronounced response. This increase in aliphatic glucosinolate concentration was probably mediated by interspecific competition resulting from root overlap of both species within intercropping and leading to an induced accumulation especially of the predominant 2-propenyl glucosinolate. Similar results have been reported by Björkman et al. (2008) on white cabbage intercropped with red clover. In contrast, when broccoli (*Brassica oleracea* var. *italica*) was grown with sesame (*Sesamum indicum*), the major aliphatic glucosinolate in broccoli, 4-methylsulphinylbutyl glucosinolate concentration was not affected by the neighboring sesame plant (Tong et al., 2015). This may indicate a certain level of specificity in plant responses to biotic stress (Kuhlmann and Müller, 2009), and the differences in the aliphatic glucosinolate formation in response to intercropping could be species-specific, or a typical structure-specific response to abiotic stress as it was found in *Brassica* (Mewis et al., 2012).

### Changes in Leaf Carotenoids and Chlorophyll Concentration

Both carotenoids and chlorophylls are largely influenced by genetic variation and growing conditions. Previous studies revealed an impact of intercropping on carotenoids as well as chlorophylls in various crops, e.g., amaranth leaves (Ng’ang’a et al., 2009), carrots (Jankowska et al., 2012), Chinese cabbage (Cai et al., 2011), and Moringa (Abou-Zeid and Salama, 2014). In our study the intercropping did not significantly change the concentration of the photosynthetic pigments, whereas the water supply impacted their concentration. While in *B. carinata* the chlorophyll b concentration was lower under water deficiency, higher levels for lutein as well as chlorophyll a and chlorophyll b have been detected under reduced water supply in *S. scabrum*. Drought induces the production of ROS (Hasegawa et al., 2000) and the lipophilic, antioxidant carotenoids help protecting membranes from damage (Havaux, 1998). Carotene accumulation in plants is consequently influenced by plant water status. It suggests that the *B. carinata* shows a higher adaptation to drought, since fewer changes have been observed in our study. Moreover, the *B. carinata* responds by accumulating specific glucosinolates, which cannot be found in *S. scabrum*. Although the influence of drought on photosynthetic pigments in vegetables has not been fully assessed yet, contrasting results have also been reported either showing a reduction or an increase of pigments, e.g., decreased chlorophyll or carotenoid concentrations have been reported in pepper (Mena-Violante et al., 2006) or chickpea (Sohrabi et al., 2012) and increased pigment concentrations in tomato (Giannakoula and Ilias, 2013). The interaction observed between drought and intercropping are an interesting finding and could be an important issue for drought stress management strategies and hence, improve water-use efficiency by constant or increased carotenoid concentrations.

### Flavonoid Diversity

The flavonoid glycosides and hydroxycinnamic acid derivative profiles of *B. carinata* and *S. scabrum* are consistent to previous studies where these compounds were identified in indigenous African vegetables (Neugart et al., 2017). It can be concluded that the profiles differ enormously between the two crops. In the present study hydroxycinnamic acids and flavonoid glycoside derivatives such as caffeic acid, coumaric acid, ferulic acid, hydroxyferulic, sinapic acid, isorhamnetin, kaempferol, and quercetin derivatives were quantified in *B. carinata* and *S. scabrum* (Nilsson et al., 2006; El-Seedi et al., 2012). Many flavonoid glycoside derivatives we identified in *B. carinata* were previously found in *B. oleracea* plants (Schmidt et al., 2010). In *B. carinata*, fifteen different flavonoid glycosides were quantified, of which, four isorhamnetin, nine kaempferol and two quercetin glycosides, with few significant differences found. The most common flavonoid glycosides found in Brassica are kaempferol glycosides (Nielsen et al., 1998; Chun et al., 2004; Mann and Khanna, 2013). However, *B. carinata* contains remarkable concentrations of isorhamnetin glycosides compared to *B. oleracea* species. Mainly kaempferol glycosides were decreased by intercropping and low irrigation. Also, on five different hydroxycinnamic acids quantified, intercropping and low irrigation had a reducing effect. These acids were found to have similar concentration in *B. carinata* plants, compared to *B. oleracea* plants (El-Seedi et al., 2012). Studies reported by Ashraf and Mehmood (1990) on selected Brassica, state that *B. carinata* is sensitive to drought stress.

From the studies done by Lewis et al. (1998), most common flavonoid derivatives found in *Solanum tuberosum* are kaempferol and quercetin glycoside, similar to that of *S. scabrum* in the present study. There were four kaempferol and ten quercetin glycosides found in *S. scabrum*, these are the most commonly found flavonoid glycosides in *Solanum* species (Lewis et al., 1998). The amount of kaempferol and quercetin glycosides were found to be more in *S. scabrum* compared to *Solanum lycopersicum* (Bovy et al., 2007). Of the eight different hydroxycinnamic acids found in *S. scabrum*, most of them were decreased with water reduction. From the studies reported by El-Seedi et al. (2012), commonly found hydroxycinnamic acids were caffeic and sinapic acids, wherein, the amount of caffeic acids were relatively similar in *S. scabrum* compared to *S. lycopersicum* and *S. melongena*. Sinapic acid was, however, identified in *S. scabrum*, and not previously identified in *S. lycopersicum* and *S. melongena*.

Mainly kaempferol glycosides and hydroxycinnamic acid derivatives of *B. carinata* and *S. scabrum* were decreased by intercropping and irrigation system. The total flavonoids of marigold and faba bean were not affected by intercropping with lettuce and maize, respectively (Li et al., 2012; Fonseca et al., 2016). However, there is evidence that the response is structure-dependent. Quercetin and kaempferol in faba bean were not affected by the intercropping with maize whereas luteolin was increased (Li et al., 2012). Referring to the results of the current study it can be suggested that a decrease of flavonoid glycosides and hydroxycinnamic acids due to intercropping is true for different species such as *B. carinata* and *S. scabrum*. The underlying mechanism is not clear yet.

In a number of species total phenolics or total flavonoids were not affected by the reduction of water by 50% (Sangtarash et al., 2009; Bernal et al., 2013). However, in a more detailed view specific hydroxycinnamic acid derivatives and quercetin as well as kaempferol glycosides are decreased due to drought in *Ligustrum vulgare* (Tattini et al., 2004), different citrus species (Zandalinas et al., 2016) and *Vitis vinifera* (Alonso et al., 2015). These species are common in the Mediterranean and other warm regions and might be adapted to drought periods during their growth. Also the plants in our experiment are used to higher temperatures and therefore the decrease in hydroxycinnamic acid derivatives and quercetin and kaempferol glycosides is consistent with the literature. André et al. (2009) demonstrated that the effect of drought on the chlorogenic acid and kaempferol-3-rutinoside of various potato cultivars is dependent on the cultivar. While three cultivars showed decreases in the before mentioned compounds one did not respond and one showed an increase. Nevertheless, the quercetin glycosides known for their higher antioxidant activity (Zietz et al., 2010) were not affected by the drought. This could explain why in the present experiment mainly hydroxycinnamic acid derivatives and kaempferol glycosides were decreased due to drought.

## Conclusion

Intercropping had no effect on biomass production or mineral nutrient uptake in both *B. carinata* and *S. scabrum* and no clear belowground competition/complementation for mineral nutrient uptake was evident under our experimental conditions. It, however, led to changes in the concentration of specific health related secondary metabolites. Intercropping *B. carinata* and *S. scabrum* led to increase in total glucosinolate content in *B. carinata* while maintaining biomass production and other health related minerals in both species. Low irrigation led to an increase in carotene accumulation in both mono and intercropped *S. scabrum*, but not in *B. carinata*. In contrast, mainly kaempferol glycosides and hydroxycinnamic acid derivatives of *B. carinata* and *S. scabrum* were decreased by intercropping and drought treatment.

This study indicates that some health-related phytochemicals can be modified by intercropping or late-season drought, however, field validation of these results is necessary before definite recommendation can be made to stakeholders.

## Author Contributions

BN: Contributed in conception and design, analysis, interpretation of data; drafting and revising content; final approval of the version to be published; agreement to be accountable for all aspects of the work. SN and SB: Contributed in conception, analysis, interpretation of data; drafting and revising content; final approval of the version to be published; agreement to be accountable for all aspects of the work. BR: Contributed in analysis, interpretation of data; revising content; final approval of the version to be published; agreement to be accountable for all aspects of the work. MS: Contributed in conception and design, interpretation of data; drafting and revising content; final approval of the version to be published; agreement to be accountable for all aspects of the work.

## Conflict of Interest Statement

The authors declare that the research was conducted in the absence of any commercial or financial relationships that could be construed as a potential conflict of interest. The reviewer ST and handling Editor declared their shared affiliation.
