# Supplementary material for: Intercropping Induces Changes in Specific Secondary Metabolite Concentration in Ethiopian Kale (Brassica carinata) and African Nightshade (Solanum scabrum) under Controlled Conditions
Source: Front Plant Sci. 2017 Sep 29;8:1700. doi: 10.3389/fpls.2017.01700 (PMC5626848; doi:10.3389/fpls.2017.01700)
Supplement: Supplementary file 1 [file Table_1.docx]

Supplementary Material

Intercropping induces changes in secondary metabolite concentration in Ethiopian kale (Brassica carinata) and African nightshade (Solanum scabrum)

**Benard Ngwene^1*^, Susanne Neugart^1^, Susanne Baldermann^1, 2^, Beena Ravi1^3^, Monika Schreiner^1^**

*** Correspondence:** Corresponding Author: [Ngwene@igzev.de](mailto:Ngwene@igzev.de)

# Supplementary Tables

|  | 3-caffeoylquinic acid | | | disinapoyl gentiobioside | | | sinapoyl-feruloyl gentiobioside | | | trisinapoyl-gentiobioside | | | disinapoyl-ferulyol gentiobioside | |
| --- | --- | --- | --- | --- | --- | --- | --- | --- | --- | --- | --- | --- | --- | --- |
| Irrigation | Mono | Mixed | | Mono | Mixed | | Mono | Mixed | | Mono | Mixed | | Mono | Mixed |
| W_opt_ | 0,053a  ±0,002 | 0,051a  ±0,001 | | 0,094a  ±0,004 | 0,092ab  ±0,008 | | 0,062  a±0,003 | 0,063a  ±0,001 | | 0,118b  ±0,005 | 0,106ab  ±0,012 | | 0,079a  ±0,003 | 0,076a  ±0,002 |
| W_low_ | 0,050a  ±0,000 | 0,050a  ±0,000 | | 0,089ab  ±0,004 | 0,083b  ±0,005 | | 0,055a  ±0,000 | 0,053a  ±0,002 | | 0,117ab  ±0,005 | 0,106a  ±0,004 | | 0,064a  ±0,001 | 0,060a  ±0,003 |
| two-way ANOVA results | | | | | | | | | | | | | | |
| Cropping (C) | ns | | ns | | | ns | | | * | | | * | | |
| Irrigation (I) | * | | * | | | * | | | ns | | | * | | |
| C x I | * | | ns | | | ns | | | ns | | | ns | | |

Table S1-A: Hydroxycinnamic acid derivative content in *B. carinata* leaves at harvest (mg/g). For treatment description, see table 1. Shown are the mean values (n=4) obtained for each treatment ± standard deviation. Values followed by the same letter are not significantly different (Tukey HSD test; P ≤ 0.05). The influence of the intercropped plant, irrigation level and the interaction between both factors estimated by a two-way ANOVA are also presented. ns: not significant; *: significant at P≤0.05

Table S1-B: Kaempferol glycosides content in *B. carinata* leaves at harvest (mg/g). For treatment description, see table 1. Shown are the mean values (n=4) obtained for each treatment ± standard deviation. Values followed by the same letter are not significantly different (Tukey HSD test; P ≤ 0.05). The influence of the intercropped plant, irrigation level and the interaction between both factors estimated by a two-way ANOVA are also presented. ns: not significant; *: significant at P≤0.05

|  | kaempferol-3-sophoroside | | | kaempferol-3-sophoroside-7-glucoside | | | kaempferol-3-coumaroyl-sophoroside-glucoside | | kaempferol-3-caffeoyl-sophoroside-7-glucoside | | kaempferol-3-feruloyl-sophoroside-7-glucoside | | | kaempferol-3-hydroxyferuoyl-sophoroside-7-glucoside | |
| --- | --- | --- | --- | --- | --- | --- | --- | --- | --- | --- | --- | --- | --- | --- | --- |
| Irrigation | Mono | | Mixed | Mono | Mixed | | Mono | Mixed | Mono | Mixed | Mono | Mixed | | Mono | Mixed |
| W_opt_ | 0,092a  ±0,008 | | 0,075a  ±0,020 | 0,507a  ±0,051 | 0,390ab  ±0,105 | | 0,043a  ±0,007 | 0,029a  ±0,008 | 0,361b  ±0,123 | 0,230ab  ±0,034 | 0,382a  ±0,071 | 0,297a  ±0,022 | | 0,646a  ±0,058 | 0,498a  ±0,106 |
| W_low_ | 0,085a  ±0,012 | | 0,068a  ±0,009 | 0,344ab  ±0,023 | 0,251b  ±0,042 | | 0,027a  ±0,011 | 0,016a  ±0,002 | 0,128ab  ±0,019 | 0,102a  ±0,023 | 0,264a  ±0,058 | 0,152a  ±0,033 | | 0,480a  ±0,017 | 0,316a  ±0,071 |
| two-way ANOVA results | | | | | | | | | | | | | | | |
| Cropping (C) | | * | | * | | * | | | * | | * | | * | | |
| Irrigation (I) | | ns | | * | | * | | | * | | * | | * | | |
| C x I | | ns | | ns | | ns | | | ns | | ns | | ns | | |

|  | | kaempferol-3-sinapoyl-sophoroside-7-glucoside | | | kaempferol-3-sinapoyl-sophoroside-7-diglucoside | | | | kaempferol-3-disinapoyl-triglucoside-7-glucoside | |
| --- | --- | --- | --- | --- | --- | --- | --- | --- | --- | --- |
| Irrigation | | Mono | Mixed | | Mono | | Mixed | | Mono | Mixed |
| W_opt_ | | 0,674a  ±0,056 | 0,528ab  ±0,116 | | 0,342a  ±0,014 | | 0,253a  ±0,034 | | 0,393a  ±0,028 | 0,319a  ±0,069 |
| W_low_ | | 0,584a  ±0,059 | 0,410b  ±0,076 | | 0,331a  ±0,023 | | 0,248a  ±0,021 | | 0,348a  ±0,025 | 0,268a  ±0,028 |
| two-way ANOVA results | | | | | |  | | | | |
| Cropping (C) | * | | | * | | | | * | | |
| Irrigation (I) | * | | | ns | | | | * | | |
| C x I | ns | | | ns | | | | ns | | |

Table S1-C: Quercetin glycosides content in *B. carinata* leaves at harvest (mg/g). For treatment description, see table 1. Shown are the mean values (n=4) obtained for each treatment ± standard deviation. Values followed by the same letter are not significantly different (Tukey HSD test; P ≤ 0.05). The influence of the intercropped plant, irrigation level and the interaction between both factors estimated by a two-way ANOVA are also presented. ns: not significant; *: significant at P≤0.05

|  | quercetin-3-O-sophoroside-7-O-D-glucoside | | | | quercetin-3-O-disinapoyl-triglucoside-7-O-D-glucoside | | isorhamnetin-3-O-sophoroside | | | | isorhamnetin-3-O-sinapoyl-sophoroside-7-O-glucoside | | | isorhanmetin-3-O-caffeoyl-sophoroside-7-O-diglucoside | | isorhamnetin-3-O-hydroxyferulyol-sophoroside-7-O-diglucoside | |
| --- | --- | --- | --- | --- | --- | --- | --- | --- | --- | --- | --- | --- | --- | --- | --- | --- | --- |
| Irrigation | Mono | | Mixed | | Mono | Mixed | Mono | | Mixed | | Mono | Mixed | | Mono | Mixed | Mono | Mixed |
| W_opt_ | 0,077a  ±0,006 | | 0,073a  ±0,020 | | 0,428a  ±0,039 | 0,399ab  ±0,077 | 0,382a  ±0,009 | | 0,377a  ±0,036 | | 0,114b  ±0,015 | 0,111ab  ±0,015 | | 0,023a  ±0,001 | 0,022a  ±0,001 | 0,052a  ±0,002 | 0,044a  ±0,009 |
| W_low_ | 0,068a  ±0,004 | | 0,059a  ±0,015 | | 0,420ab  ±0,049 | 0,383b  ±0,034 | 0,458a  ±0,105 | | 0,371a  ±0,014 | | 0,115ab  ±0,014 | 0,090a  ±0,021 | | 0,022a  ±0,005 | 0,018a  ±0,001 | 0,063a  ±0,008 | 0,052a  ±0,009 |
| two-way ANOVA results | | | | | | | | | | | | | | | | | |
| Cropping (C) | | ns | | ns | | | | ns | | ns | | | * | | | * | |
| Irrigation (I) | | ns | | ns | | | | ns | | ns | | | * | | | * | |
| C x I | | ns | | ns | | | | ns | | ns | | | ns | | | ns | |

Table S1-D: Hydroxycinnamic acid derivative content in *S. scabrum* leaves at harvest (mg/g). For treatment description, see table 1. Shown are the mean values (n=4) obtained for each treatment ± standard deviation. Values followed by the same letter are not significantly different (Tukey HSD test; P ≤ 0.05). The influence of the intercropped plant, irrigation level and the interaction between both factors estimated by a two-way ANOVA are also presented. ns: not significant; *: significant at P≤0.05

|  | | 3-caffeoylquinic acid | | | | | | | | 5-caffeoylquinic acid | | | | 4-caffeoylquinic acid | | | | | | caffeoylmalate | | | | |
| --- | --- | --- | --- | --- | --- | --- | --- | --- | --- | --- | --- | --- | --- | --- | --- | --- | --- | --- | --- | --- | --- | --- | --- | --- |
| Irrigation | | Mono | | | | | Mixed | | | Mono | Mixed | | | Mono | | | Mixed | | | Mono | | Mixed | | |
| W_opt_ | | 0.122a  ±0.018 | | | | | 0.118a  ±0.028 | | | 0,087a  ±0,009 | 0,084ab  ±0,026 | | | 0,638a  ±0,041 | | | 0,525a  ±0,163 | | | 0,101b  ±0,013 | | 0,102ab  ±0,026 | | |
| W_low_ | | 0.094a  ±0,004 | | | | | 0.082a  ±0,010 | | | 0,070ab  ±0,003 | 0,062b  ±0,004 | | | 0,380a  ±0,036 | | | 0,256a  ±0,057 | | | 0,084ab  ±0,006 | | 0,070a  ±0,009 | | |
| two-way ANOVA results | | | | | | | | | | | | | | | | | | | | | | | | |
| Cropping (C) | | | | ns | | | | | ns | | | | * | | | | | ns | | | | | | |
| Irrigation (I) | | | | * | | | | | * | | | | * | | | | | * | | | | | | |
| C x I | | | | ns | | | | | ns | | | | ns | | | | | ns | | | | | | |
|  | | | Caffeoylmalate | | | | | | | coumaric acid | | | | | sinapoylmalate | | | | | | sinapic acid | | |  |
| Irrigation | | Mono | | | Mixed | | | | Mono | Mixed | | | | Mono | Mixed | | | | | Mono | | Mixed |  |  |
| W_opt_ | | | 3,250a  ±0,183 | | | 2,983a  ±0,787 | | | | 0,036a  ±0,002 | 0,033a  ±0,005 | | | | 0,065a  ±0,003 | 0,062a  ±0,004 | | | | | 0,209a  ±0,024 | | 0,193a  ±0,069 |  |
| W_low_ | | | 2,543a  ±0,197 | | | 2,257a  ±0,184 | | | | 0,031a  ±0,002 | 0,031a  ±0,004 | | | | 0,060a  ±0,001 | 0,058a  ±0,001 | | | | | 0,157a  ±0,010 | | 0,138a  ±0,023 |  |
| two-way ANOVA results | | | | | | | | | | | | | | | | | | | | | | | |  |
| Cropping (C) | | | | | ns | | | ns | | | | ns | | | | | | | ns | | | | |  |
| Irrigation (I) | | | | | * | | | ns | | | | * | | | | | | | * | | | | |  |
| C x I | | | | | ns | | | ns | | | | ns | | | | | | | ns | | | | |  |

Table S1-E: Kaempferol glycoside content in *S. scabrum* leaves at harvest (mg/g). For treatment description, see table 1. Shown are the mean values (n=4) obtained for each treatment ± standard deviation. Values followed by the same letter are not significantly different (Tukey HSD test; P ≤ 0.05). The influence of the intercropped plant, irrigation level and the interaction between both factors estimated by a two-way ANOVA are also presented. ns: not significant; *: significant at P≤0.05

|  | kaempferol-3-diglucoside | | | kaempferol-3-glucosylrhamnogalcatoside | | | kaempferol-3-rhamnosylrhamnogalactoside (isomer 1) | | | kaempferol-3-rhamnosylrhamnogalactoside (isomer 2) | |  |
| --- | --- | --- | --- | --- | --- | --- | --- | --- | --- | --- | --- | --- |
| Irrigation | Mono | Mixed | | Mono | Mixed | | Mono | Mixed | | Mono | Mixed | |
| W_opt_ | 0,057a  ±0,005 | 0,056a  ±0,007 | | 0,043a  ±0,003 | 0,041ab  ±0,003 | | 0,083a  ±0,007 | 0,079a  ±0,023 | | 0,040b  ±0,001 | 0,039ab  ±0,004 | |
| W_low_ | 0,051a  ±0,003 | 0,050a  ±0,003 | | 0,040ab  ±0,001 | 0,040b  ±0,003 | | 0,074a  ±0,010 | 0,060a  ±0,012 | | 0,038ab  ±0,001 | 0,038a  ±0,002 | |
| two-way ANOVA results | | | | | | | | | | | |  |
| Cropping (C) | ns | | ns | | | ns | | | ns | | |  |
| Irrigation (I) | * | | ns | | | ns | | | ns | | |  |
| C x I | ns | | ns | | | ns | | | ns | | |  |

|  | quercetin-3-neohesperidoside-7-glucorahmnoside | | | | quercetin-3-rutinoside-7-rahmnoglucoside | | | quercetin-3-galactorhamnoside | | | quercetin-3-rhamnogalactoside | | | quercetin-3-pentosylglucoside | | | quercetin-3-pentosylrutinoside | |
| --- | --- | --- | --- | --- | --- | --- | --- | --- | --- | --- | --- | --- | --- | --- | --- | --- | --- | --- |
| Irrigation | Mono | | Mixed | | Mono | Mixed | | Mono | Mixed | | Mono | Mixed | | Mono | Mixed | | Mono | Mixed |
| W_opt_ | 0,085a  ±0,008 | | 0,085a  ±0,021 | | 0,260a  ±0,025 | 0,261ab  ±0,081 | | 0,049a  ±0,007 | 0,052a  ±0,013 | | 0,064b  ±0,027 | 0,036ab  ±0,015 | | 0,022a  ±0,000 | 0,021a  ±0,001 | | 0,235a  ±0,028 | 0,209a  ±0,040 |
| W_low_ | 0,065a  ±0,002 | | 0,062a  ±0,013 | | 0,177ab  ±0,006 | 0,158b  ±0,052 | | 0,039a  ±0,007 | 0,042a  ±0,007 | | 0,035ab  ±0,009 | 0,034a  ±0,011 | | 0,022a  ±0,000 | 0,021a  ±0,001 | | 0,195a  ±0,009 | 0,154a  ±0,056 |
| two-way ANOVA results | | | | | | | | | | | | | | | | | | |
| Cropping (C) | | ns | | ns | | | ns | | | ns | | | * | | | ns | | |
| Irrigation (I) | | * | | * | | | * | | | ns | | | ns | | | * | | |
| C x I | | ns | | ns | | | ns | | | ns | | | ns | | | ns | | |

Table S1-F : Quercetin glycoside content in *S. scabrum* leaves at harvest (mg/g). For treatment description, see table 1. Shown are the mean values (n=4) obtained for each treatment ± standard deviation. Values followed by the same letter are not significantly different (Tukey HSD test; P ≤ 0.05). The influence of the intercropped plant, irrigation level and the interaction between both factors estimated by a two-way ANOVA are also presented. ns: not significant; *: significant at P≤0.05

|  | quercetin-3-rhamnosylrhamnogalactoside (isomer 1) | | | | quercetin-3-rhamnosylrhamnogalactoside (isomer 2) | | | quercetin-3-rhamnosylrhamnogalactoside (isomer 3) | | | quercetin-3-glucosylrhamnogalcatoside | |
| --- | --- | --- | --- | --- | --- | --- | --- | --- | --- | --- | --- | --- |
| Irrigation | Mono | | Mixed | | Mono | Mixed | | Mono | Mixed | | Mono | Mixed |
| W_opt_ | 0,023a  ±0,001 | | 0,022a  ±0,001 | | 0,244a  ±0,027 | 0,206ab  ±0,020 | | 0,433a  ±0,058 | 0,405a  ±0,095 | | 0,313b  ±0,058 | 0,294ab  ±0,097 |
| W_low_ | 0,023a  ±0,001 | | 0,022a  ±0,001 | | 0,182ab  ±0,013 | 0,129b  ±0,042 | | 0,314a  ±0,029 | 0,268a  ±0,102 | | 0,233ab  ±0,042 | 0,215a  ±0,027 |
| two-way ANOVA results | | | | | | | | | | | | |
| Cropping (C) | | ns | | * | | | ns | | | ns | | |
| Irrigation (I) | | ns | | * | | | * | | | * | | |
| C x I | | ns | | ns | | | ns | | | ns | | |
